# Merging Mixture Components for Cell Population Identification in Flow Cytometry

**DOI:** 10.1155/2009/247646

**Published:** 2009-11-12

**Authors:** Greg Finak, Ali Bashashati, Ryan Brinkman, Raphaël Gottardo

**Affiliations:** ^1^Computational Biology Unit, Clinical Research Institute of Montreal, 110 Pine Avenue West, Montreal, QC, Canada H2W1R7; ^2^Terry Fox Laboratory, BC Cancer Research Center, Vancouver, BC, Canada V5Z 1L3; ^3^Département de Biochimie, Université de Montreal, Montreal, QC, Canada

## Abstract

We present a framework for the identification of cell subpopulations in flow cytometry data based on merging mixture components using the flowClust methodology. We show that the cluster merging algorithm under our framework improves model fit and provides a better estimate of the number of distinct cell subpopulations than either Gaussian mixture models or flowClust, especially for complicated flow cytometry data distributions. Our framework allows the automated selection of the number of distinct cell subpopulations and we are able to identify cases where the algorithm fails, thus making it suitable for application in a high throughput FCM analysis pipeline. Furthermore, we demonstrate a method for summarizing complex merged cell subpopulations in a simple manner that integrates with the existing flowClust framework and enables downstream data analysis. We demonstrate the performance of our framework on simulated and real FCM data. The software is available in the flowMerge package through the Bioconductor project.

## 1. Introduction

Flow cytometry (FCM) can be applied in a high-throughput fashion to process thousands of samples per day. However, data analysis can be a significant challenge because each data set is a multiparametric description of millions of individual cells. Consequently, despite widespread use, FCM has not reached its full potential due to the lack of an automated analysis platform to assist high-throughput data generation. 

A critical bottleneck in data analysis is gating, the identification of groups of similar cells for further study. The process involves identification of regions in multivariate space containing homogeneous cell populations of interest. Generally, gating has been performed manually by expert users, but manual gating is subject to user variability, which can potentially impact results [1–3].

A number of methods have been developed to automate the gating process [4–7]. These include model-based methods such as multivariate mixture models that describe the joint density of the flow cytometry data as a mixture of simpler distributions [5, 6]. The simplest of these methods utilizes a mixture of multivariate gaussian distributions [5]. However it is not sufficiently flexible to model the outliers or asymmetrical cell populations frequently found in flow cytometry data [6].

A more recent approach compensates for these effects by applying a data transformation during the model fitting process [6, 8]. This transformation makes data more symmetric, while the use of a multivariate *t* distribution allows the model to handle outliers [6, 8, 9].

These model-based gating methods effectively amount to clustering of the data and generally employ likelihood-based measures such as the Bayesian information criterion (BIC) or Akaike information criterion (AIC) to select an appropriate model (number of clusters) from a range of possibilities [10]. While these measures are effective for choosing a model that provides a good fit to the underlying data distribution, they are problematic for clustering flow cytometry data, where the goal is to determine the correct number of distinct cell populations. BIC favors models with more mixture components in order to provide a better fit to the data distribution [11]. However, this comes at the cost of overestimating the number of well-separated clusters, particularly when clusters are asymmetric and/or nonconvex.

An alternative measure recently proposed for model selection is the Integrated Complete Likelihood (ICL)[11]. The ICL is an entropy-penalized BIC criterion, wherein the BIC is penalized by an entropy term, which increases as a function of the overlap between model components. Consequently, ICL favors models with fewer components and provides a better estimate of the number of well-separated populations; however this generally comes at the cost of a poor fit to the empirical data distribution, especially if clusters are asymmetric, nonconvex, or otherwise not readily fit by a simple parametric distribution [12].

In flow cytometry, where the shapes of cell populations can be asymmetric and nonconvex, neither of the above model fitting criteria are well suited to the clustering problem. An ideal model would allow multiple mixture components to represent an individual cluster or cell population, thus providing a good fit to the data and a good estimate of the number of distinct clusters. Such an algorithm has recently been proposed for Gaussian mixture models (GMMs) [12]. The algorithm starts with the best model selected by the BIC criterion and iteratively merges pairs of overlapping clusters in order to minimize the entropy of the model [12]. Because it is based on the best fitting BIC model, this approach retains the good distributional fitting properties of the best BIC model, while simultaneously allowing multiple mixture components to represent a single cluster. Like the ICL measure, it also provides a reasonable estimate of the number of well separated clusters in the data [12]. Merging clusters to improve fitting of nonconvex cell population has also recently been suggested by Pyne et al. [13].

Here we extend the work of Baudry et al. to subpopulation identification in flow cytometry data [12]. We combine the cluster merging algorithm with the more flexible model classes provided by a multivariate *t*-mixture with Box-Cox transformed data and develop a method for summarizing merged clusters that is compatible with the flowClust framework [6]. Additionally, we automate the choice of the number of clusters in the cluster merging algorithm, making it suitable for application in a high throughput FCM analysis pipeline. We propose a method for the identification of borderline cases where the merging algorithm fails, which can be flagged for manual analysis. In Table 1 we list the distributional assumptions, model selection criteria, and the abbreviations used to refer to the five models compared throughout this paper.

Employing the cluster merging algorithm under the flowClust framework provides a better fit and a better estimate of the number of distinct cell populations for complicated flow cytometry data distributions, than either the flowClust_BIC_, flowClust_ICL_, GMM_BIC_, or GMM_ICL_ models. The cluster merging algorithm provides a simpler visual representation of the data that is more amenable to interpretation. We demonstrate the performance of our algorithm on simulated and real FCM data. The software is available through the Bioconductor project. 

## 2. Materials and Methods

### 2.1. The flowClust Framework

We embed the cluster merging algorithm within the flowClust framework available in BioConductor [6, 14]. The flowClust package is used to fit mixture models of multivariate *t* distributions to flow cytometry data. Additionally, the model allows the data to be Box-Cox transformed during model fitting, with the goal of making the data distribution more symmetric and bringing it closer to “normality”. The model allows a number of parameters to be estimated from the data, including the degrees of freedom *ν* of the multivariate *t* distributions being fitted and the Box-Cox transformation parameters *λ* (Table 1). While flowClust does allow independent degrees of freedom and independent Box-Cox transformation parameters to be estimated for each mixture component, we chose to use a common degrees of freedom and common Box-Cox transformation parameter, estimated from the data, across all mixture components in a model. This was done in order to have closed form estimates of summary statistics for the merged components. Note also that this additional flexibility is not necessary in our framework as subpopulations can be represented as mixtures of multiple components. In the rest of this paper, we refer to this as the *flowClust* model.

### 2.2. The Cluster Merging Algorithm

We have implemented the cluster merging algorithm described in [12], with several modifications allowing its use with flow cytometry data within the flowClust framework. Briefly, we begin with the optimal flowClust_BIC_ solution of *K* clusters. At the first iteration of the algorithm, two clusters are chosen for merging in order to minimize the entropy of the data under the new cluster assignments, as described in [12]. The entropy of clustering for a *K* cluster mixture model is defined as

(1) $$\begin{array}{c} \text{ENT}\left(K\right)=-2\overset{K}{\underset{k=1}{\sum }}\;\overset{N}{\underset{i=1}{\sum }}p_{ik}{\log\;}_{2}\left(p_{ik}\right) \end{array},$$

where *p*_*i k*_ is the probability of data point *i* belonging to cluster *k*. Thus for two overlapping clusters *k*, *k* + 1, the probability of a data point *i* in the overlapping region belonging to either cluster is nonzero, and the entropy is high. If the clusters overlap very little or not at all, then the entropy is zero or near zero. Consequently, by iteratively merging overlapping components, the entropy of clustering is reduced. At each successive iteration, two more clusters are merged until, at the *K*th iteration, the data is defined by a single cluster.

Baudry et al. suggest two data-driven approaches for choosing the optimal *k*-cluster solution [12]. The first involves identifying an “elbow” in a plot of the entropy of clustering versus the number of clusters in a solution. The second involves identifying peaks in a plot of the number of clusters versus the change in entropy obtained by merging two clusters in the *k* + 1 cluster solution into a single cluster to form the *k* cluster solution (see [12] for details). Here, we propose an automated approach for choosing the optimal *k*-cluster solution based on changepoint analysis of the entropy versus number of clusters plot, making the cluster merging algorithm suitable for inclusion in an automated workflow for flow cytometry data analysis [8].

### 2.3. Parameter Representation of Merged Mixture Components

It is important to be able to have a parametric representation of merged clusters in order to summarize characteristics of the population. To this end, we model a merged cluster as a multivariate *t* distribution with degrees of freedom, *ν*, equal to the degrees of freedom of its component clusters. We let **X**_*i*_ and **X**_*j*_ be random variables that represent the *p* dimensional measurements of cells in clusters *i* and *j*. We let X_*_ be the random variable that represents the *p* dimensional measurements of cells in the cluster created by merging clusters *i* and *j* (i.e., any two clusters). We let f_*_, *f*_*i*_, and *f*_*j*_ be the distributions of X_*_, **X**_*i*_, and **X**_*j*_, respectively, and *n*_*i*_, *n*_*j*_ the number of events in clusters *i* and *j*, respectively. Thus f_*_ can be written as a mixture of *f*_*i*_ and *f*_*j*_ (see [12] for details) as follows:

(2) $$\begin{array}{c} p_{*}f_{*}=p_{i}f_{i}+p_{j}f_{j}. \end{array}$$

Thus, by definition, the proportion of cells p_*_ in the merged cluster is equal to the sum of the proportions of the components *p*_*i*_ and *p*_*j*_, given by

(3) $$\begin{array}{c} p_{*}=p_{i}+p_{j}. \end{array}$$

Because we model the merged cluster as a single multivariate *t* distribution we can summarize merged components with individual sets of parameters describing their locations and scales. To estimate the mean and covariance matrix of the merged component, we match the first two moments of the distributions in (2) (see [15]), giving

(4) $$\begin{array}{cc} \mu_{*} & =\frac{\left(p_{i}\mu_{i}+p_{j}\mu_{j}\right)}{p_{*}},\\ {\text{Σ}}_{*} & =\frac{\left(\nu_{*}-2\right)p_{i}\left[\left(\nu_{i}/\left(\nu_{i}-2\right)\right){\text{Σ}}_{i}+\mu_{i}\mu_{i}^{\prime }\right]}{p_{*}\nu_{*}}\\ & \;+\frac{\left(\nu_{*}-2\right)p_{j}\left[\left(\nu_{j}/\left(\nu_{j}-2\right)\right){\text{Σ}}_{j}+\mu_{j}\mu_{j}^{\prime }\right]}{p_{*}\nu_{*}}\\ & \;-\frac{\left(\nu_{*}-2\right)p_{*}\mu_{*}\mu_{*}^{\prime }}{p_{*}\nu_{*}}. \end{array}$$

The expressions in (4) are the mean vector and covariance matrix of the merged distribution, which is approximated by a multivariate *t* model with ν_*_ = *ν*_*i*_ and *ν*_*i*_ = *ν*_*j*_ degrees of freedom. As previously mentioned, a common Box-Cox transformation parameter allows us to estimate the parameters of the merged clusters on the transformed scale.

### 2.4. Estimating the Number of Clusters/Cell Subpopulations

Our stopping criteria for merging are based on analysis of the number of clusters in a solution versus the clustering entropy of that solution. Intuitively, when mixture components overlap significantly, the entropy of clustering will be a large value. As components are combined in subsequent iterations of the merging algorithm, the entropy will decrease. When only well separated components are left in the clustering solution, further merging will have little impact on the total entropy of clustering. This is reflected in a change of slope in the plot of the clustering entropy versus the number of components at the point, where the remaining clusters are well separated. We refer to this as the optimal flowMerge solution.

We formalize this idea by fitting piecewise linear regression to the entropy versus the number of clusters in the series of flowMerge model and allow the regression to have either one or two segments (i.e., one or no changepoint). Furthermore, we force the location of the changepoint to be an integer, thus reflecting the discrete nature of the clustering. Formally, if we have *K* models with an increasing number (1 ⋯ *K*) clusters, we fit a series of two-segment piecewise linear regressions to the entropy versus the number of clusters in the mixture models. The first segment is fit to the data points for mixture models 1 ⋯ *k* and the second segment to the data points for models *k* ⋯ *K*, where *k* ∈ {2 ⋯ *K* − 1}, assuming *K* > 3. The position of the change point, *k*, is chosen to minimize the residual sum of squares between the observed data and the piecewise regression line. Once we have selected the location of the changepoint, we choose between the presence and absence of a changepoint (i.e., two-segment piecewise regression versus simple linear regression) using the BIC criterion.

When *K* = 3, there are not enough data points to fit a changepoint model, therefore we determine the presence or absence of a changepoint by computing the angle *θ* between the two component regression lines, given by *θ* = arctan(|*a* − *b*|/(1 + *a b*))(180/*π*) where *a* and *b* are the slopes of the two lines. We set an empirical cutoff of *θ* = 1 degree for identification of a changepoint. Another borderline case is for *K* = 2 clusters, in which case we always return the two component solution. For these borderline cases, the sample is flagged with a warning. In practice, however, we have rarely found cases where the flowClust_BIC_ fit has *K* < 4 components.

### 2.5. Identifying Borderline Cases

We flag potential cases where the merging algorithm fails to identify a good solution through several different criteria.

1. If the number of clusters in the flowMerge solution is equal to the number of clusters in the flowClust_BIC_ solution.
2. If the number of clusters in the flowMerge solution is less than the number of clusters in the flowClust_ICL_ solution.
3. If no changepoint is detected (BIC chooses no change point model).
4. If the entropy of the flowMerge solution is unusually high (an outlier) compared to the entropy of the flowMerge solution for comparable samples using the same markers.

In the above cases, samples are flagged for manual inspection of the automated gating. To facilitate the comparison in (4), we normalize the entropy by the number of events in the sample as well as the number of clusters in the merged solution:

(5) $$\begin{array}{c} \text{EN}{\text{T}}_{N}\left(K\right)=\frac{-2\sum_{k=1}^{K}\sum_{i=1}^{N}p_{ik}{\log\;}_{2}\left(p_{ik}\right)}{NK}. \end{array}$$

### 2.6. The CLL Data Set

We applied the cluster merging algorithm to a real-world data set consisting of 137 samples from 18 individuals with CLL (chronic lymphocytic leukemia) provided by the BC Cancer Agency. The data set is composed of between six and seven samples per individual. Each sample is labeled with three fluorescent markers. The entire panel of markers is designed for immunophenotyping of lymphomas in a clinical setting (Table 2). 

We performed automated gating using flowClust on the forward scatter and side scatter channels, followed by cluster merging of the optimal flowClust_BIC_ solution. We compared the number of clusters obtained by the flowClust_BIC_, flowClust_ICL_, and flowMerge solutions. The lymphocyte subpopulation was selected from the merged solution and automated gating was applied to this subpopulation in the fluorescence dimensions. Again, the flowClust_BIC_, flowClust_ICL_, and flowMerge solutions were compared, as well as the GMM_BIC_ solution.

### 2.7. Simulation

We simulated data from the empirical distribution of a real FCM data set. Based on the CD8 versus CD4 projection of a CLL sample, we estimated the empirical distribution using a two-dimensional kernel density estimator on a 100 by 100 point grid, and sampled 100 data sets of size *N* = 9198 equal to the original number of events. Events were simulated in a two-step process, first we sampled according to the CD8 marginal density derived from the two-dimensional kernel density estimate on a 100 × 100 point grid, then sampled in the CD4 dimension, conditional on the sampled CD8 value, defined by the 100 × 1 element bin of the kernel density estimate. The simulated data sets were gated using the manual gates established on the original data for CD8+/CD4−, CD8−/CD4+, and CD8−/CD4− cell populations (Figure 6(a)). These manual gates were used to calculate misclassification rates for automated gating using the flowClust_BIC_, flowClust_ICL_, flowMerge_K_, and GMM_BIC_ models with the number of clusters fixed at the true number (*K* = 3) and with the number of clusters chosen by the optimal model.

## 3. Results

### 3.1. CLL Data Set

**Figure 1:**
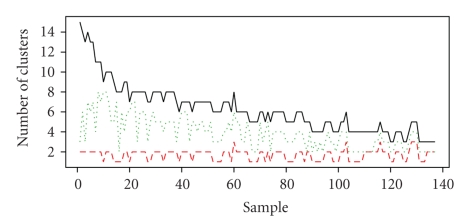
flowClust_BIC_, flowClust_ICL_, flowMerge solutions for automated gating of forward versus side scatter across 137 clinical samples of CLL. The flowClust_BIC_ fit: black solid curve. The flowClust_ICL_ fit: red dashed curve. The flowMerge fit: green dashed curve.

**Figure 2:**
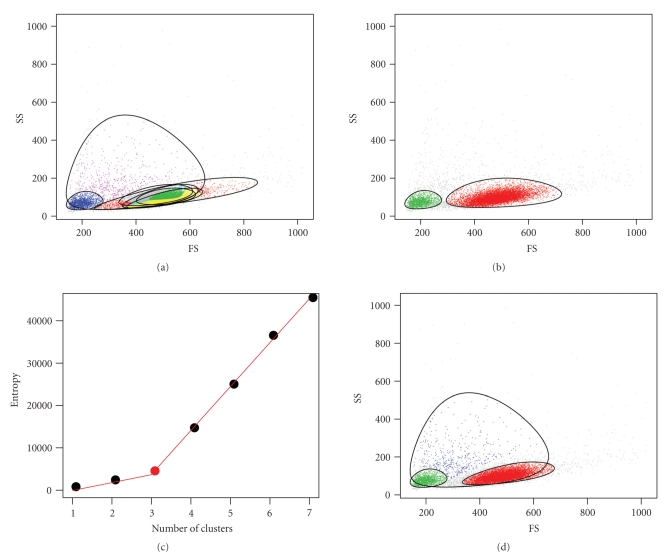
Examples of the flowClust_BIC_, flowClust_ICL_, flowMerge cluster solutions for forward versus side scatter in a sample of CLL flow cytometry data. (a) The flowClust_BIC_ solution with seven clusters. (b) The flowClust_ICL_ solution with two clusters. (c) The entropy versus number of clusters plot, fit to a two-component piecewise linear regression model. The best fitting model has a changepoint at three clusters. (d) The flowMerge solution corresponding to *K* = 3 clusters provides a better fit to the lymphocyte population than either the flowClust_BIC_ or flowClust_ICL_ solutions and provides a good estimate of the true number of cell populations.

We compared the number of clusters identified by the flowClust_BIC_, flowClust_ICL_, flowMerge models used for automated gating of 137 lymph node-derived CLL samples in the forward versus side scatter dimensions (Figure 1). The forward and side scatter data for these samples contain between two and three predominant cell populations that correspond to lymphocytes, debris, and outliers. The number of clusters identified by the flowClust_BIC_ solution shows large variability across all samples. This solution generally required more mixture components than the true number of cell populations (median 6 clusters, range 3–15). Importantly, multiple components were often required to model the lymphocyte population (Figure 2(a)), which is the cell population of interest.

In contrast, the flowClust_ICL_ fit is better but tends to underestimate the true number of cell populations. Across the 137 CLL samples, ICL identified a median of two populations per sample (range from 1 to 3). The ICL also provides a poor fit to the data, inadequately modeling the lymphocyte population (Figure 2(b)).

The flowMerge solution derived from the flowClust_BIC_ solution provides both a good fit to the underlying data, including the lymphocyte cell population, as well as an improved estimate of the true number of cell populations (Figures 2(c) and 2(d)). The number of clusters estimated through merging is generally between the flowClust_BIC_ and flowClust_ICL_ solutions (median of 4 populations, range 2 to 8 clusters).

**Figure 3:**
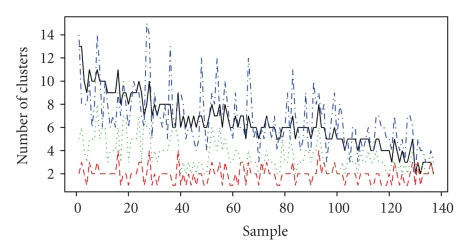
The number of clusters chosen by the flowClust_BIC_, flowClust_ICL_, flowMerge, and GMM_BIC_ solutions for automated gating of CD8, CD4, and CD7 across 137 samples of CLL. The flowClust_BIC_ solution: solid black curve. The flowClust_ICL_ solution: dashed red curve. The flowMerge solution derived from the flowClust_BIC_ solution: dashed green curve. The GMM_BIC_ solution: dashed blue curve.

We performed automated gating in the fluorescence channels on the lymphocyte subpopulation derived from the previous autogating step. In 60/137 cases (43%), the GMM_BIC_ solution returned more clusters than the flowClust_BIC_ solution. In 95% of those cases the GMM_BIC_ fit was within 5 components of the flowClust_BIC_ fit. These two models returned an equal number of clusters in 29/137 cases (21%), and in 48/137 (35%) of cases, the GMM_BIC_ fit had fewer components. However, in the latter cases, 95% of the samples differed by only a single component (Figure 3, black curve). In general, for the fluorescence dimensions, the flowClust_BIC_ model estimated fewer cell subpopulations than the GMM_BIC_ model, in accordance with what is expected, given that the former is a more robust and flexible model.

**Figure 4:**
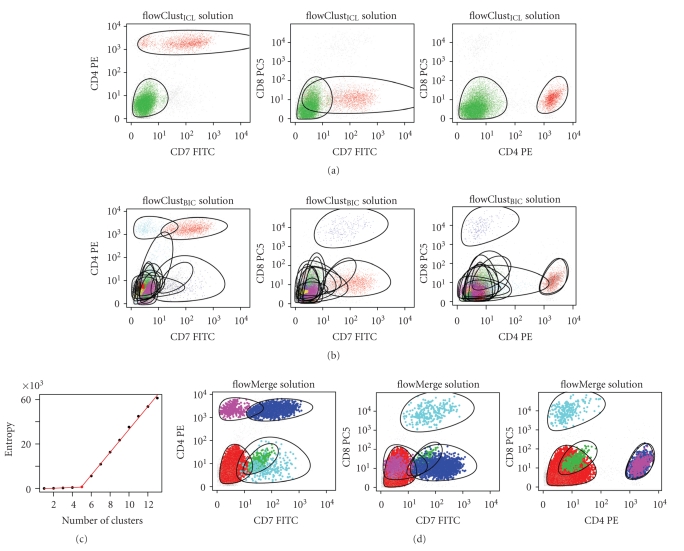
Example of flowClust_ICL_, flowClust_BIC_, and flowMerge solutions fitted to a CLL sample in the CD8, CD4, and CD7 dimensions. (a) Three projections of the flowClust_ICL_ solution. (b) Three projections of the flowClust_BIC_ solution. (c) Entropy versus number of clusters for a series of flowMerge model fits with a piecewise linear regression fitted to the data. The changepoint located at *K* = 5 clusters is selected automatically. (d) Three projections of flowMerge solution with *K* = 5 clusters derived from the flowClust_BIC_ solution.

The flowClust_ICL_ fit generally underestimated the number of cell subpopulations and provided a poor fit to the data distribution (Figure 3, red curve and Figure 4(a)). In the example shown, the flowClust_ICL_ solution identifies two cell subpopulations in the CD8/CD4/CD7 dimensions and fails to discriminate between the CD4+/CD7+ and CD4+/CD7− cell subpopulations. Additionally, it entirely fails to capture the CD8+ cell subpopulation (Figure 4(a)).

In contrast, for the same sample, the flowClust_BIC_ fit requires 13 components and clearly overestimates the number of cell subpopulations. Specifically, the CD4−/CD7−/CD8− cells require multiple mixture components to model a single subpopulation (Figure 4(b)).

The choice of the number of clusters for the flowMerge solution is automated by fitting a piecewise linear model to the entropy versus number of clusters (Figure 4(c)). This solution is derived from the flowClust_BIC_ fit and provides a good compromise between model fit and subpopulation identification. It correctly discriminates between the different unique cell subpopulations that were missed by the flowClust_ICL_ solution, while combining the overlapping mixture components required to model the CD8/CD4/CD7 negative cell subpopulation in the flowClust_BIC_ solution (Figure 4(d)).

**Figure 5:**
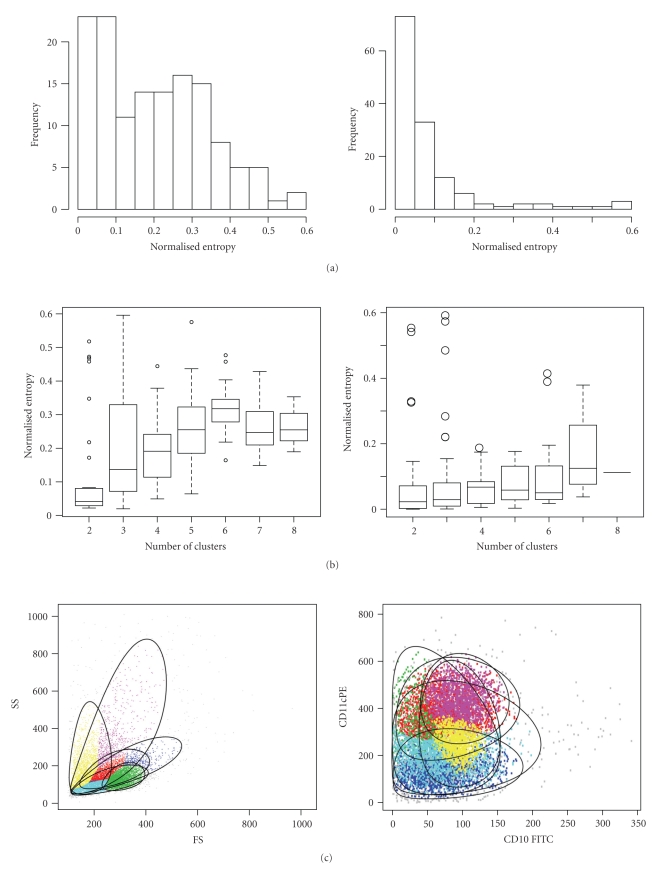

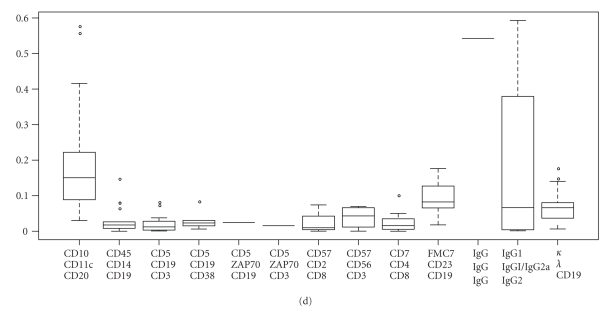
Detecting failed cluster merging. (a) Distribution of the entropy (normalized for the number of events and clusters) of the flowMerge solution for forward versus side scatter (left) and fluorescence channels (right) across 137 samples. (b) The relationship between the normalized entropy and the number of clusters in the flowMerge solution for forward scatter versus side scatter (left) and fluorescence channels (right). (c) Example of flowMerge solutions with unusually high normalized entropy from the right tail of the distribution for forward versus side scatter (left) and fluorescence (right). (d) A plot of the normalized entropy versus samples grouped by antibody labels identifies antibody combinations that are problematic for automated gating with the automated merging algorithm.

We identify cases where cluster merging fails by examining the distribution of the entropy of the flowMerge solution across multiple comparable samples (Figures 5(a)–5(d)). In the forward versus side scatter dimensions, cell populations tend to be complex and overlapping. This is reflected in the distribution of the normalized entropy (Figure 5(a), left). The normalized entropy of the merged solution has a broad distribution (90% of the samples below 0.4, median 0.2) and the solution itself may have many clusters. In contrast, for the fluorescence dimensions, the merged solution identifies well separated populations, reflected by a normalized entropy distribution that is tightly distributed around zero (90% of samples below 0.2, median 0.03) (Figure 5(a), right). We correct for the relationship between the entropy and the number of clusters in the merged solution as well as the number of events by normalizing the entropy (Figure 5(b)). Normalization reduces the correlation of the entropy with the number of clusters (*ρ* = 0.38 versus *ρ* = 0.77 for FS versus SS, and *ρ* = 0.08 versus *ρ* = 0.49 for fluorescence dimensions) (Figure 5(b)). This allows us to identify flowMerge solutions where the entropy is unusually large (in the right tail of the distribution), independent of the number of clusters or events. For forward versus side scatter and for fluorescence channels, we can identify samples where the merged solution contains highly overlapping components (Figure 5(c)). None the less, for forward versus side scatter, the lymphocyte population is sufficiently dense that it can be readily identified visually. Such cases are therefore flagged for manual analysis. Importantly, this criterion allows us to identify general classes of samples where merging fails. We note several sets of markers (notably CD10/CD11c/CD20 and Kappa/Lambda/CD19), where the normalized entropy of clustering is high for all, or a majority of samples (Figure 5(d)). This type of outlier detection is suitable for a high throughput setting to quickly assess flowMerge model fit across groups of parameters and identify those where the automated merging algorithm is problematic. In these cases, again, manual inspection may be required to find an appropriate merged solution. More careful analysis of these cases could suggest strategies to improve automated gating techniques for flow cytometry data.

### 3.2. Simulation

**Figure 6:**
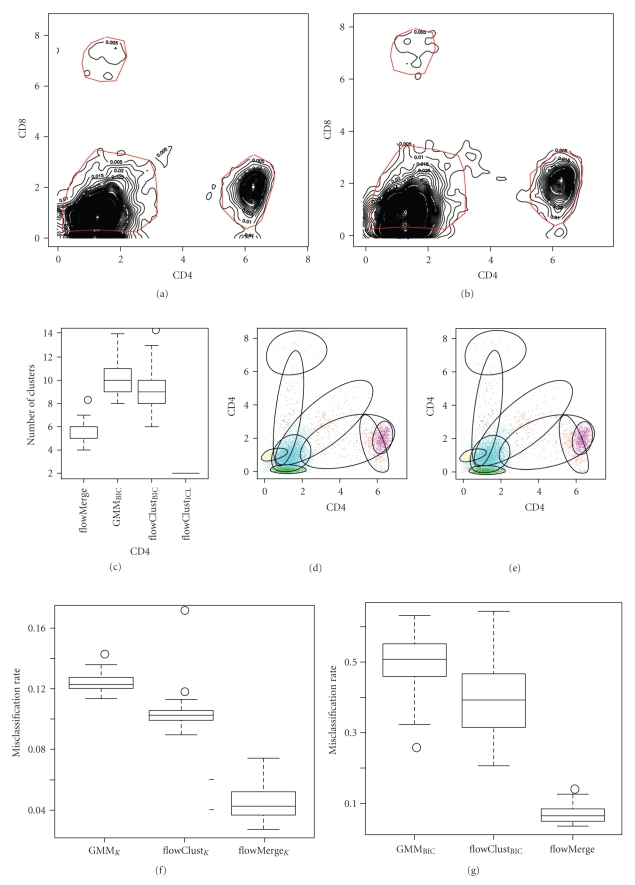

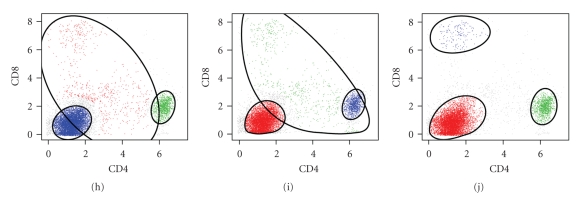
Simulation results for CD4 versus CD8 dimensions of a CLL sample. (a) The 2D kernel density estimate of the real CD4 versus CD8 data. Gates for the CD4+/CD8− , CD8+/CD4− , and CD4−/CD8− subpopulations are represented by light coloured lines. Events outside the gates are considered outliers. (b) An example of the kernel density estimate of simulated data drawn from the distribution defined by the real data. (c) The number of clusters selected by the flowMerge solution, the GMM_BIC_ solution, the flowClust_BIC_, and flowClust_ICL_ solutions over 100 realizations of simulated data. (d) The median flowClust_BIC_ flowClust solution with 9 components. (e) The median flowMerge solution with 5 components. (f) The misclassification rate (MCR) for the flowMerge_K_ solution, the GMM_K_ solution, and the flowClust_K_ solution with the number of clusters fixed to the true number of cell subpopulations (*K* = 3). (g) The misclassification rates for the three components from the optimal GMM_BIC_, flowClust_BIC_, and flowMerge solutions minimizing the MCR. (h) A GMM, (i) flowClust, (j) and flowMerge_K_ solution with a fixed number of clusters.

We simulated 100 data sets of CD8 versus CD4 fluorescence based on the empirical distribution of real CD8 versus CD4 CLL data. This simulation approach ensured that the simulated data was not biased towards any of the models under investigation. This data had three cell subpopulations defined based on the contours in the CD4 versus CD8 dimensions. These included CD4+/CD8− cells, CD8+/CD4− cells, CD4−/CD8− cells, (outliers were defined by events outside these gates) (Figure 6(a)). No CD4+/CD8+ cell subpopulation could be discerned from the kernel density estimate of this particular sample. We simulated 9198 events per sample (equal to the number of events in the original data) and assigned them to populations based on the manually defined gates from the original data. Kernel density estimates based on simulated data are comparable to the original data (Figure 6(b)).

We compared the number of clusters selected under the optimal flowClust_ICL_, flowClust_BIC_, GMM_BIC_, and flowMerge solutions (Figure 6(c)). The flowClust_ICL_ solution systematically underestimated the true number of subpopulations (2 clusters estimated in all simulations). The GMM_BIC_ and flowClust_BIC_ solutions both significantly overestimated the true number of cell subpopulations in all simulations (median 10 and 9, resp., Figure 6(c)). The median flowClust_BIC_solution (*K* = 9 clusters, Figure 6(d)) required two components to model the CD4+/CD8− subpopulation, one for the CD8+/CD4− subpopulation, three for the CD4−/CD8− subpopulation, and three components for modeling various outlier low-frequency subpopulations. Although the flowMerge solution overestimated the true number of clusters on average, it provided the closest estimate of the true number of cell subpopulations (median 5). In 16% of simulations, the flowMerge solution estimated the correct number of clusters. In 51% of simulations it overestimated the true number by only one cluster. Closer examination reveals that the extra clusters serve predominantly to model outlier populations (Figure 6(e)). These results are summarized in Table 3.

We also compared the misclassification rates for the different models, relative to class assignments from manual gating. This was done in two ways. First, we fixed the number of clusters to the true number (*K* = 3) for the flowClust_K_, GMM_K_, and flowMerge_K_ models (Figure 6(f)). Note that the former three sets of models are distinct from their “optimal” counterparts by virtue of fixing the number of clusters. Alternately, we compute the misclassification rate between the optimal flowClust_BIC_, flowMerge or GMM_BIC_ solutions, choosing the three components from each that minimize the misclassification rate (Figure 6(g)). When the number of components was fixed to the true number, the GMM_K_ model had the highest misclassification rate (12.3%) (Figure 6(h)), flowClust_K_ had the second highest misclassification rate (10.5%) (Figure 6(i)), while the flowMerge_K_ solution (with fixed *K*) derived from the optimal flowClust_BIC_ model, had the lowest misclassification rate (4.2%) (Figure 6(j) and Table 3). Both the GMM_K_ and the flowClust_K_ solutions with a fixed number of components failed to correctly identify the rare CD8+/CD4− cell subpopulation in the simulated data (Figures 6(h) and 6(i)). In contrast, the flowMerge_K_ solution correctly identified this subpopulation as a distinct entity.

The misclassification rates for the optimal flowClust_BIC_, flowMerge, and GMM_BIC_ solutions were calculated as described, relative to the manually derived gates (Figure 6(g)). These followed a pattern similar to the misclassification rates with a fixed number of components (GMM_BIC_ was the highest, followed by flowClust_BIC_, followed by flowMerge). However, in contrast to the fixed component solutions, the misclassification rates for the flowClust_BIC_ and GMM_BIC_ solutions were significantly higher than the flowMerge solution (Table 3). This is due to the fact that multiple model components are required to represent distinct cell populations, something only permitted within the cluster merging framework.

## 4. Discussion

Model-based automated gating of flow cytometry data is difficult when cell subpopulations are nonconvex, or have complicated multidimensional shapes that are not readily modeled by single components of simpler multivariate distributions. This issue is resolved, in part, by allowing multiple mixture components to represent the same cell subpopulation. However, for further analysis, cell subpopulations are generally summarized by a variety of statistics; this requires one to summarize an arbitrary number of mixture components for a single cell subpopulation. Consequently the cluster merging algorithm is not suitable for application to flow cytometry data without further modifications. By taking advantage of the fact that a merged cluster is itself a mixture (see (2)), and approximating the merged distribution as a density from the same family as its components, we use moment matching to summarize the merged cluster with a single set of parameters that provides a good approximation to the underlying data (see (3) and (4)). This simple representation of otherwise complicated distributions allows downstream data analysis to proceed in the usual manner and fits within the existing flowClust framework, allowing for easy visualization of automated gating results.

Comparison of the cluster merging algorithm with other automated gating models (Table 1) using both simulated and real data demonstrate that merging provides a better fit and better estimate of the true number of cell subpopulations than the other models. Estimates of the number of cell populations derived from standard model-selection measures such as BIC or ICL are not entirely suitable for flow cytometry data (Figures 2 and 4). BIC, while providing a good fit to the data, requires many more clusters than actual number of cell subpopulations, while ICL underestimates the number of cell subpopulations and provides a poor fit to the data, missing both rare cell subpopulations and poorly fitting those that have complicated structure (Figures 4(a), 4(b) and Table 3). The flowMerge solution provides a good compromise between these two extremes. It is based on the flowClust_BIC_ solution, thus retaining the property of good fit to the distribution, while simultaneously eliminating ambiguity associated with multiple overlapping components representing the same cell subpopulation. Merging decreases the entropy of clustering by making local changes to the model without compromising the global fit. 

We use a changepoint model to estimate the optimal number of clusters in the merged solution. This allows the cluster merging algorithm to be implemented in a high-throughput pipeline for flow cytometry data analysis. In general, this approach provides satisfactory results, both for forward versus side scatter dimensions as well as for fluorescence dimensions (Figures 1 and 3). The number of clusters chosen by flowMerge is generally between the flowClust_BIC_ and flowClust_ICL_ solutions, and although it still tends to overestimate the number of cell subpopulations by several components, these generally model outlier cell subpopulations (Figure 2(d) and 6(e)). Interestingly, our simulation results also show that our framework for summarizing merged components allows some of these outlier subpopulations to be merged with clusters representing more dense cell subpopulations, of interest, without adversely affecting the fit of the model. This is due to the fact that the parameters of merged clusters are weighted linear combinations of the parameters of the component clusters. Therefore components of lower density contribute less to the mean and covariance parameters of merged clusters (Figures 6(e)–6(g)). 

Our results on real flow data demonstrate that the cluster merging algorithm improves our ability to identify the lymphocyte cell subpopulation from the forward versus side scatter dimensions. This high density subpopulation is often represented by multiple mixture components in the flowClust_BIC_ and GMM_BIC_ solutions. Merging allows this subpopulation to be represented by a single model component (Figure 2). Even in cases where merging fails, the algorithm is sufficiently robust that prior information about the expected number of cell populations could be used to identify an appropriate merged solution manually, while retaining a good fit to the data distribution (Figures 6(d) and 6(j)). Others have suggested incorporating information from the repeated-measures design of some flow cytometry data sets to help make gating decisions [16]. The application of cluster merging for identification of cell populations in the fluorescence dimensions is also beneficial. It reduces the complexity of subpopulations represented by multiple components. A comparison of the flowClust_BIC_ and flowClust_ICL_ solutions shows that these two criteria tradeoff model fit against a simpler representation of cell subpopulations (Figures 4(a) and 4(b)). The flowClust_ICL_ solution frequently fails to correctly identify all but the highest density regions; whereas the flowClust_BIC_ solution often overestimates the number of clusters in high density regions.

Our cluster merging framework provides a robust modeling approach for automated gating of flow cytometry data. It provides a good compromise between the flowClust_BIC_ and flowClust_ICL_ solutions by combining the good model fitting characteristics of BIC-based model selection with a more modest estimate of the true number of clusters, a characteristic of the ICL-based model selection. It allows us to represent complicated cell populations using single mixture components for which we can readily obtain closed-form parameter estimates for use in further analysis. Additionally, these estimates are robust to outlier cell populations. The cluster merging approach to gating has a lower misclassification rate than other models considered here, irrespective of whether the number of clusters was fixed at the true number or chosen from amongst the components in the optimal fitting model. Together, these factors make cluster merging a powerful tool for automated gating of flow cytometry data.

## Figures and Tables

**Table 1:** Distributional assumptions, data transformation, and model selection criteria for the five clustering models compared in this study.

Distribution	Transformation	Model selection criteria	Model name
Multivariate-*t*	Box-Cox	BIC	flowClust_BIC_
	Box-Cox	ICL	flowClust_ICL_
	Box-Cox	Fixed K	flowClust_K_
	Box-Cox	BIC, entropy	flowMerge
	Box-Cox	BIC, entropy, fixed K	flowMerge_K_

Gaussian	None	BIC	GMM_BIC_
	None	ICL	GMM_ICL_
	None	fixed K	GMM_K_

**Table 2:** Summary of the antibody markers used in the CLL data.

Antibody combination	Ab1	Ab2	Ab3	No. tubes
1	CD10	CD11	CD20	18
2	CD45	CD14	CD19	18
3	CD5	CD19	CD3	18
4	CD5	CD19	CD38	5
5	CD5	ZAP70	CD19	1
6	CD5	ZAP70	CD3	1
7	CD57	CD2	CD8	4
8	CD57	CD56	CD3	4
9	CD7	CD4	CD8	13
10	FMC7	CD23	CD19	18
11	IgG	IgG	IgG	1
12	IgG1	IgG1/IgG2a	IgG2	13
13	Kappa	Lambda	CD19	18

**Table 3:** Mean, standard deviation, 95% coverage, and bias of the estimated number of clusters for each model, as well as the mean, standard deviation and 95% coverage for the misclassification rate of each model. CI: coverage interval.

Statistic	Model	Mean	SD	95% CI	Bias
Number of clusters	flowClust _BIC_	9.03	1.59	6–12	6.03
	flowClust_ICL_	2.00	—	2-2	−1.00
	GMM_BIC_	10.41	1.31	8–12	7.14
	flowMerge	5.45	0.97	4–7	2.45

Misclassification rate (*K* = 3)	flowClust	0.103	0.00826	0.0937–0.112	—
	GMM	0.124	0.00537	0.114–0.134	—
	flowMerge_K_	0.0445	0.0104	0.0312–0.0669	—

Misclassification rate (best model)	flowClust_BIC_	0.398	0.101	0.230–0.613	—
	GMMBIC	0.499	0.0756	0.339–0.625	—
	flowMerge	0.0685	0.0223	0.0383–0.121	—
